# Ultraviolet – Chlorine combined treatment efficiency to eliminate *Naegleria fowleri* in artificial surf lagoons

**DOI:** 10.1016/j.heliyon.2022.e11625

**Published:** 2022-11-18

**Authors:** Iñigo Arberas-Jiménez, Ines Sifaoui, María Reyes-Batlle, Aitor Rizo-Liendo, Luis Sancho, Andoni Urruticoechea, José E. Piñero, Jacob Lorenzo-Morales

**Affiliations:** a Instituto Universitario de Enfermedades Tropicales y Salud Pública de Canarias (IUETSPC), Universidad de La Laguna (ULL), Avenida Astrofísico Francisco Sánchez s/n, 38206, La Laguna, Tenerife, Spain; b Departamento de Obstetricia y Ginecología, Pediatría, Medicina Preventiva y Salud Pública, Toxicología, Medicina Legal y Forense y Parasitología, Universidad de La Laguna, Tenerife, Spain; c Red de Investigación Cooperativa en Enfermedades Tropicales (RICET), Spain; d CEIT and Tecnun (University of Navarra) Manuel Lardizabal 15, 20018, Donostia, San Sebastián, Spain; e Wavegarden, Instant Sport S.L, Spain; f Consorcio Centro de Investigación Biomédica (CIBER) de Enfermedades Infecciosas (CIBERINFEC), Instituto de Salud Carlos III, 28006, Madrid, Spain

**Keywords:** Free-living amoeba, *Naegleria fowleri*, cove lagoon, UV light, Chlorination, Disinfection

## Abstract

*Naegleria*. fowleri, a protozoa belonging to the free-living amoeba group, is the causative agent of a central nervous system affecting disease that is fatal in more than the 95% of the reported cases. This parasite can be found in warm water bodies such as lakes, rivers or inadequately disinfected swimming pools. On the other hand, chlorination and UV light treatment are two of the most extensively used disinfection methods in recreational water facilities.

In this study the effect of chlorination and UV light on *N. fowleri* trophozoites was studied in a close water circuit with the aim to assess the efficacy of this disinfection methods in large pools. The obtained results showed that the chlorination was able to decrease the number of viable cells despite the elimination was not totally achieved. Nonetheless, the combination of the UV light with the chlorination allowed the complete removal of the *N. fowleri* trophozoites from the water in experimental testing conditions.

## Highlights

• Absence of an standardized water treatment protocol to eliminate *N. fowleri.*
• Treatment based only on chlorine was ineffective to eliminate *N. fowleri.*
• Combined chlorination and UV light treatment was effective to completely eliminate *N.fowleri*.
• Combination of chlorine - Uv would be a promising method for water disinfection.

## Graphical abstract

**Figure:**
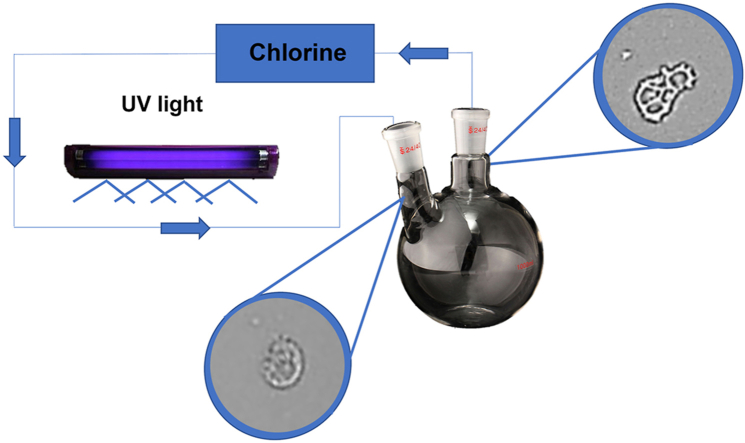

## 1 Introduction

Waters from rivers, lakes and costal used for recreational purposes are classified as recreational water (RW). RW are widely used by people for all manner of recreational activities, including swimming, surfing, white water sports, diving, boating and fishing. Furthermore, those activities were proved to have substantial benefits to health and well-being [1]. Artificial lagoons are nowadays some of the biggest RW bodies existing in the world and companies need to guarantee a safe environment for people enjoying perfect surf waves. Actually, RW users can be exposed to a wide range of microorganisms namely those introduced through human or animal faecal contamination or those naturally present in water, that are found mostly in polluted and unsafe environments [2]. Water's free-living microorganisms are autochthonous to water or once introduced are capable of colonizing the environment [1].

Among those microorganisms, Free-Living Amoebae (FLA) are widespread in nature and are normal inhabitants of freshwater microbial ecosystems, soil, water and artificial habitats as swimming pools [3, 4, 5]. FLA are considered opportunistic pathogens. In fact, and based on the literature, *Acanthamoeba* spp, *Naegleria fowleri*, *Balamuthia mandrillaris*, *Vahlkampfia, Vermamoeba* and *Sappinia* infections have already been reported in humans as well as in animals. On the other hand, FLA can also act as hosts and vectors of pathogenic micro-organisms as bacteria or virus [6]. Only *Acanthamoeba* spp. and *N. fowleri* were cited and defined by WHO as quality indicator for non-faecal in recreational water. *Naegleria* genus are amoebo-flagellates and not as ubiquitous as *Acanthamoeba*. It was isolated particularly from warm freshwater bodies including manmade lakes and ponds, hot springs, and thermally polluted streams and rivers [7]. Currently, more than 40 species of *Naegleria* have been reported but only one species, *N. fowleri*, is pathogenic to humans. The pathogenicity of two other species have been proved in mice after intranasal or intracerebral inoculations [7].

Outbreaks of recreational water illness have been linked to poor system design and lack in the water treatments process [8]. In order to prevent the spreading of illnesses in RW the pathogen should remain under control and to do so RW should undergo several treatments including coagulation, filtration, disinfection [8].

Disinfection is a fundamental step in water treatment process by which a microbial hazard is eliminated or inactivated [8, 9]. During this treatment chemical (e.g. chlorination) or physical (e.g. filtration, UV radiation) agent are used to deactivate the pathogen [1]. Chlorination is the most extensively used disinfectant for water treatments due to its low cost, ease to produce, store, transport and use as well as its high oxidizing potential. Usually used as chlorine gas, sodium or calcium hypochlorite, it provides a minimum level of residual disinfectant able to prevent microbial recontamination [10]. Chlorine dioxide have been as well reported by Dupuy et al., (2014) for its efficacy to inhibit three different FLA strains [11]. UV radiation can decrease the microbial charge in the air, on hard surfaces and in thin layer of liquid food and thus by microbial deactivation. It can also eliminate pathogens from potable water and fruit juices [12]. In fact, Yip & Konasewich, (1972) have proved that the UV radiation was effective to eliminate various microorganisms including viruses, bacterial spores and protozoa [13]. Since then, the disinfection of drinking water and wastewater by UV light was investigated in several studies [14].

Artificial surfing waves lagoons consist of large water bodies where huge amounts of water circulate along a closed circuit and in which there are man made waves. However, no specific protocol is available for the FLA treatment and disinfection of this kind of pools. In the present study, the efficacy of Chlorination and UV radiation treatments separated or combined was conducted against *N. fowleri*. To assess the efficiency of disinfection methods tested an experimental device to reproduce large pools systems are designed. The cells were analyzed during the time using inverted microscopy. Technologies and operation conditions (free chlorine concentration, Contact Time (CT), UV doses) applied in this study are in the range of the operational conditions of these variables applied in artificial lagoons water management system and though, experimental results and conclusions could be extrapolated to real facilities.

## 2 Material and methods

### 2.1 The used amoeba strains

The disinfection methods were evaluated against the type strain of *N. fowleri* (ATCC 30808™) of the American Type Culture Collection (LG Promochem, Barcelona, Spain). The amoebae were axenically cultured at 37 °C in 2% Bactocasitone medium (Thermo Fisher Scientific, Madrid, Spain) supplemented with 10% (v/v) of foetal bovine serum (FBS), 0.5 mg/ml of streptomycin sulphate and 0.3 μg/ml of penicillin G (Sigma-Aldrich, Madrid, Spain). This *N. fowleri* strain was cultured in a biological security facility level 3 at the Instituto Universitario de Enfermedades Tropicales y Salud Pública de Canarias, Universidad de La Laguna as required by the Spanish Government biosafety guidelines for this pathogen.

### 2.2 Experimental set-up

**Figure 1:**
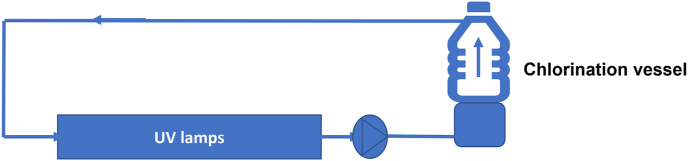
Experimental setup for chlorination and UV treatment.

A recirculating system represented in Figure 1 was set-up to carry out batch test. The system was equipped with UV lamps to provide. Actual UV doses was measured with an UV254 Lightmeter and adjusted to doses in the same range as used in WaveGarden Cove facilities as shown in Table 1. Chlorine was introduced in the chamber. The chlorine concentration was measured using the 4500-Cl G DPD method (APHA, 2005) Contact time adopted was adjusted to similar values as in the WaveGarden Cove facilities. In the same way, UV doses were applied using an UVC lamp in a holding quartz chamber to provide UV doses in the range of WaveGarden Cove facilities.

**Table 1:** Physical-chemical conditions in the present experiment compared to standard disinfection conditions in surf lagoon.

S	Standard disinfection conditions	Conditions for Chlorine test	Conditions for combined UV + chlorine test
Recirculation frequency (hours)	65	0.108	0.027
UV dose (mW/cm2.sec)	60	60	60
Chlorine dose in pipe (CT) (mg/L·min)	60	52.48	52.48
Residual chlorine	0.25	ND	ND

### 2.3 Water pump infection protocol

Initially, the system was filled with 1600 ml of filtered (0.22 μm filter) tap water and the pump was activated, with a flow rate of 150 rpm, so the water was distributed through the circuit. A total of 10^6^ amoebae were introduced in the pool with agitation. After 10 min, a sample was collected as negative control. To measure the concentration, 15 ml of water were taken from the pool and then centrifugated to concentrate the amoebae in 100 μl. The counting was carried out in a Neubauer chamber following the manufacturer protocol to obtain the amoebae concentration in the pool. Gibco™ Trypan Blue Solution, 0.4% (Thermo Fisher Scientific, Madrid, Spain) was used to distinguish between viable and non-viable cells.

### 2.4 Amoebicidal effect evaluation after the water chlorination in a close circuit

To evaluate the effect of the water chlorination against the selected amoebae strain, chlorine was added obtaining a final concentration of 2 mg/L in the chlorination vessel. Finally, samples were collected at 5 and 10 min to measure the number of cells using the same protocol than in the negative control.

### 2.5 Amoebicidal effect evaluation of the ultraviolet light in a close water circuit

The negative control and the pump filling were performed in the same manner than the chlorination assay. Therefore, the UV lamp was turned on and the water was maintained circulating for 10 min. Afterwards, another sample was collected to finally calculate the cells concentration as it was described in the water pump infection protocol.

### 2.6 Water chlorination and UV light combination assay

Firstly, the water was chlorinated at a final concentration in the vessel of 2 mg/L and maintained in the chlorination vessel to the defined CT value, afterwards the UV lamp was turned on and the recirculation maintained during 10 min. Finally, a water sample was taken, and the amoebae concentration was calculated as previously described in the 2.3 section.

## 3 Results

### 3.1 Amoebicidal effect after water chlorination in a close water circuit

**Figure 2:**
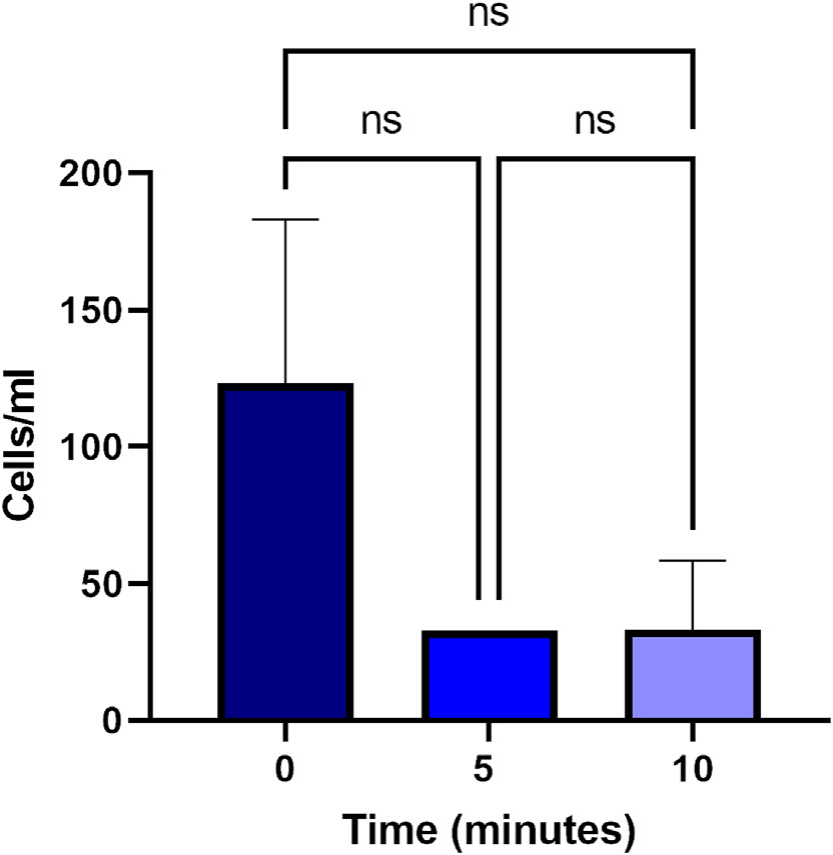
*N. fowleri* counting after 5 and 10 min of chlorination. Data are represented as means ± standard deviation. Differences between the values were assessed using one-way analysis of variance (ANOVA). Ns: not significant.

After the amoeba inoculation, a total of 15 mL of water samples from the pool were concentrated after 10 min without treatment as a negative control. The sample was concentrated up to 100 μL to the double counting in the Neubauer chamber (10^3^ cells/mL). After the addition of chlorine, we could observe a decrease in the number of cells (Figure 2). However, this reduction in the number of viable trophozoites was not significant and we were still able to count alive trophozoites.

These results are in accordance with the few other studies that compare these disinfectants on various genera of FLA [11, 15].

### 3.2 Amoebicidal effect of the UV light in a close water circuit

The negative control was measuring as it was previously described for the UV effect, obtaining a total of 291 cells/mL after dilution/concentration corrections. Subsequently, the UV assay was performed for 10 min to achieve a 60 mW/cm·sec UV dose, and 15 mL were used for the Neubauer amoebae counting. After 10 min of UV treatment, there were only nonviable amoebae trophozoites in the analysed samples.

### 3.3 Amoebicidal effect after the combination of UV light and chlorination in a close water circuit

**Figure 3:**
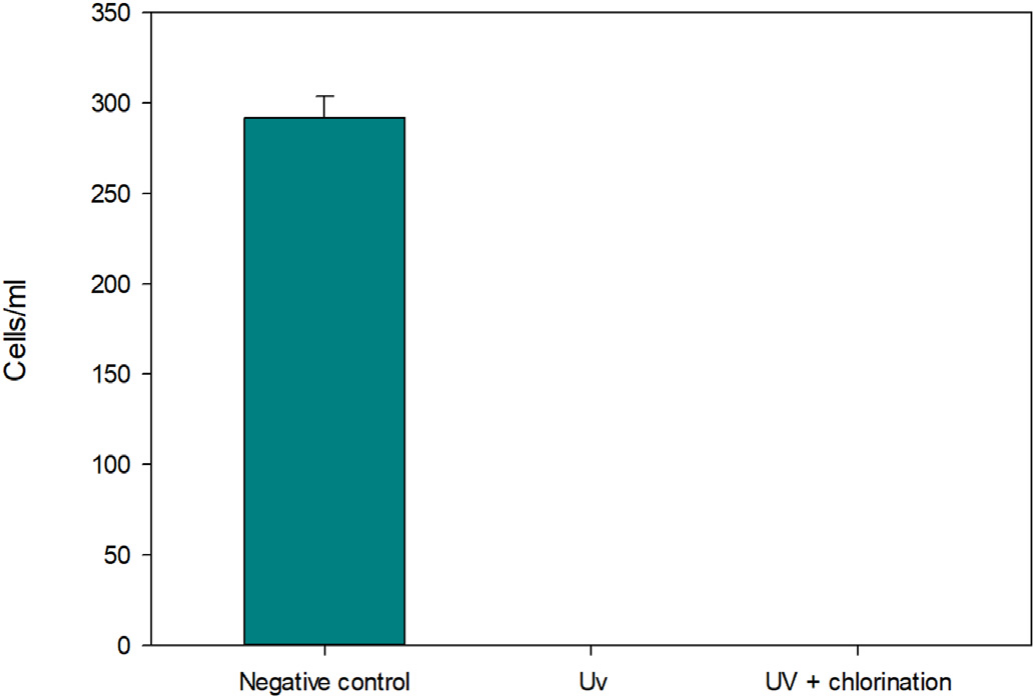
*N. fowleri* viable cells counting after the UV light treatment and after the combination of UV light and chlorination. Data are represented as means ± standard deviation.

To increase the efficacy of the disinfection method, we decide to combine both UV and chlorination protocols. Therefore, a CT of 52 mg/L·min was applied in the chlorination vessel after that an UV light were applied for 10 min of treatment to complete the 60 mW/cm·sec dose. As a result, we could observe the non-viable cells resulting from this combined treatment (Figure 3).

## 4 Discussion

*N. fowleri,* is an opportunistic parasite which causes a severe and acute meningoencephalitis in people who reported previous water exposure. Therefore, since various cases have been recently reported in water parks [16], indoor swimming pools [17] or surfing parks [18], the disinfection and maintenance of recreational water facilities are being submitted to a more exhaustive analysis.

In this context, the disinfection and maintenance of recreational water facilities has gained special attention, particularly since various cases have been recently reported in water parks [16], indoor swimming pools [17] or surfing parks [18].

UV light and chlorination are two of the most widely used disinfection methods to treat swimming pools and recreational water facilities. Moreover, the combination of both techniques is not only more efficient when killing microorganisms but it is also safer for the swimming pool users since the formation of chlorination by-products is significantly reduced [19, 20]. In fact, the chlorination alone is not able to eliminate some pathogenic microorganisms, such as *Cryptosporidium parvum* or *Giardia lambia* [21]. Because of these reasons, the interest in alternative disinfecting methods has increased, been the UV light one of the most popular among them. The UV irradiation inactivates microorganisms by inducing a variety of mutagenic and cytotoxic DNA lesions such as cyclobutane pyrimidine dimers and 6-4 photoproducts formation, as well as DNA strand breaks by interfering the genome integrity [22, 23], therefore it does not produce any harmful chemical products [24].

The first part of the assay consisted on evaluating the potential of the chlorination (at a final concentration of 2 mg/ml) of the water to eliminate the *N. fowleri* trophozoites. As it can be illustrated on Figure 2, after 5 min of treatment the number of viable trophozoites was reduced whereas it was not significant. Moreover, the decrease of cells stopped after this time and after 10 min of treatment the number of amoebae was similar, suggesting that the chlorine has an amoebostatic activity. Several studies have reported that the *N. fowleri* trophozoites are fairly resistant to chlorination [25, 26]. Moreover, the pipe walls of the RW facilities usually contain biofilms which increase the resistance of *N. fowleri* to chlorination due to the disinfectants consumption by the biofilm and the reduced disinfectant penetration into the biofilm [27].

On the other hand, when treating the water with both UV light and chlorine, the elimination of viable *N. fowleri* cells was fully effective showing no viable cells in the circuit. Furthermore, the UV dose administered in this study (60 mW s/cm^2^) was also effective against the resistant phase (cyst stage) of *N. fowleri* according to Sarkar & Gerba [25]. These results demonstrate the great effectiveness of the UV light application in water disinfection, which has been increased in this assay with the chlorination. However, the limitations of the UV light in certain circumstances should also be considered [28]. In recent studies, the control and elimination of organisms by using different nanoparticles has also been reported, so it may be possible to use these new technologies to remove free-living amoebae in aquatic environments too [29, 30, 31].

## 5 Conclusions

This paper analyses the effect of two disinfection technologies, chlorination and UV radiation, commonly used in recreational waters to remove *N. fowleri*. The results show significant *N. fowleri* removal with chlorine doses applied but viable cells still appear in treated water. The use of UV and their combination with similar doses of chlorine achieves a complete *N. fowleri* removal in the experimental testing conditions.

## Declarations

### Author contribution statement

Iñigo Arberas-Jiménez; Ines Sifaoui; María Reyes-Batlle; Aitor Rizo-Liendo: Performed the experiments; Analyzed and interpreted the data; Contributed reagents, materials, analysis tools or data; Wrote the paper.

Luis Sancho; Andoni Urruticoechea: Conceived and designed the experiments; Wrote the paper.

José Piñero; Jacob Lorenzo-Morales: Conceived and designed the experiments; Performed the experiments; Contributed reagents, materials, analysis tools or data; Wrote the pape.

### Funding statement

This work was funded by projects PI18/01380 from Instituto de Salud Carlos III, Spain and RICET (RD16/0027/0001 project) and PID2019-109476RB-C21 (BIOALGRI) (Spanish Ministry of Science, Madrid, Spain; from Programa Redes Temáticas de Investigación Cooperativa, FIS (Ministerio Español de Salud, Madrid, Spain) and FEDER. Consorcio Centro de Investigación Biomédica (CIBER) de Enfermedades Infecciosas (CIBERINFEC), Instituto de Salud Carlos III, 28006 Madrid, Spain. IAJ (TESIS202001063) and ARL (TESIS2020010054) were funded by Agencia Canaria de Investigación, Innovación y Sociedad de la Información (ACIISI).

### Data availability statement

Data will be made available on request.

### Declaration of interest's statement

The authors declare no conflict of interest.

### Additional information

No additional information is available for this paper.
